# β‐Lactoglobulin affects the oxidative status and viability of equine endometrial progenitor cells via lncRNA‐mRNA‐miRNA regulatory associations

**DOI:** 10.1111/jcmm.17694

**Published:** 2023-03-01

**Authors:** Krzysztof Data, Klaudia Marcinkowska, Klaudia Buś, Lukas Valihrach, Edyta Pawlak, Agnieszka Śmieszek

**Affiliations:** ^1^ Department of Experimental Biology, The Faculty of Biology and Animal Science University of Environmental and Life Sciences Wroclaw Poland; ^2^ Laboratory of Gene Expression, Institute of Biotechnology CAS, Biocev Vestec Czech Republic; ^3^ Laboratory of Immunopathology, Department of Experimental Therapy Hirszfeld Institute of Immunology and Experimental Therapy Wroclaw Poland

**Keywords:** β‐lactoglobulin, endometrium, non‐coding RNA, progenitor cells

## Abstract

The β‐lactoglobulin (β‐LG) was previously characterized as a mild antioxidant modulating cell viability. However, its biological action regarding endometrial stromal cell cytophysiology and function has never been considered. In this study, we investigated the influence of β‐LG on the cellular status of equine endometrial progenitor cells under oxidative stress. The study showed that β‐LG decreased the intracellular accumulation of reactive oxygen species, simultaneously ameliorating cell viability and exerting an anti‐apoptotic effect. However, at the transcriptional level, the reduced mRNA expression of pro‐apoptotic factors (i.e. BAX and BAD) was accompanied by decreased expression of mRNA for anti‐apoptotic BCL‐2 and genes coding antioxidant enzymes (CAT, SOD‐1, GPx). Still, we have also noted the positive effect of β‐LG on the expression profile of transcripts involved in endometrial viability and receptivity, including ITGB1, ENPP3, TUNAR and miR‐19b‐3p. Finally, the expression of master factors of endometrial decidualization, namely prolactin and IGFBP1, was increased in response to β‐LG, while non‐coding RNAs (ncRNAs), that is lncRNA MALAT1 and miR‐200b‐3p, were upregulated. Our findings indicate a novel potential role of β‐LG as a molecule regulating endometrial tissue functionality, promoting viability and normalizing the oxidative status of endometrial progenitor cells. The possible mechanism of β‐LG action includes the activation of ncRNAs essential for tissue regeneration, such as lncRNA MALAT‐1/TUNAR and miR‐19b‐3p/miR‐200b‐3p.

## 1 INTRODUCTION

β‐Lactoglobulin (β‐LG) is a small globular protein consisting of 162 amino acid residues that belong to lipocalins, which are potential regulators of cellular communication.^1^, ^2^, ^3^, ^4^ The β‐LG has an antioxidative activity, but its potential does not exceed that of vitamin E and probucol.^2^ Moreover, β‐LG is an efficient transporter of molecules with antioxidant and anticancer properties, including phenolic compounds, vitamins and many others.^1^, ^5^, ^6^, ^7^, ^8^

The β‐LG is a major component of ruminant milk of most species, except for humans, guinea pigs, rabbits and rodents. Thus, the biological role of β‐LG is considered regarding its nutritional profile and/or allergenicity.^3^, ^4^ However, since β‐LG degrades into smaller peptides, which undergo self‐assembly to form structures that mimic the extracellular microenvironment of many cell types, it can also find comprehensive biomedical application in regenerative medicine. For instance, fibrils formed from whey protein isolate containing β‐LG supported the metabolic activity of human bone marrow stromal cells (hBMSC) and promoted their attachment and spreading, eventually facilitating functional differentiation into bone‐forming cells.^5^ Furthermore, β‐LG fibrils functionalized with chitosan‐formed biomimetic nanotopographies improved the adipogenic differentiation of hBMSCs.^6^

When deliberating the bioactivity of β‐LG, we also paid attention to the homology with human glycodelin (Gd), which participates in the metabolic activity and differentiation of the human endometrial cells.^1^, ^7^ Given that β‐LG modulates the differentiation of progenitor cells^9^, ^10^ and may affect their oxidative status, we were interested in whether it will influence the metabolism of endometrial progenitor cells.

Indeed, the oxidative status of the endometrial progenitor cells significantly impacts their functional differentiation and cellular plasticity. Previously, we have shown that obesity‐induced oxidative stress affects the metabolism of equine endometrial progenitor cells (Eca EPCs), decreasing the dynamics of the mitochondrial network and deteriorating the regenerative potential of cells related to proliferative activity and multipotency maintenance.^8^ Moreover, oxidative stress impairs the decidualization of endometrial stromal cells (ECSs), inducing their apoptosis and declining mitochondrial membrane potential.^11^ On the contrary, mounting evidence indicates that resistance to oxidative stress and intracellular reactive oxygen species (ROS) levels increase during the decidualization of ESCs.^9^, ^10^

Decidualization of EPCs is essential to assuring embryo implantation, thus a prerequisite for a successful pregnancy. This process is governed by ovarian hormones, that is oestrogen and progesterone, that cooperate in regulating pregnancy and menstruation.^9^ Decidualization of endometrial stromal cells is associated with cytophysiological changes in endometrial stromal cells, related to their transformation into specialized secretory decidual cells characterized by expression of phenotypic markers, that is prolactin (PRL) and insulin‐like growth factor‐binding protein‐1 (IGFBP‐1).^9^, ^12^ In addition, the involvement of non‐coding RNAs (ncRNAs) in the process of decidualization is now intensively studied to reveal novel molecular pathways and find their physiological and clinical relevance.^13^ Besides elements of the PRL axis, other molecular markers gain importance as factors essential for proper embryo implantation and blastocyst attachment. For example, ectonucleotide pyrophosphatase‐phosphodiesterase 3 (ENPP3) undergoes cyclic changes in the endometrium regulating its receptivity,^14^ while lncRNA TUNAR (TCL1 upstream neural differentiation‐associated RNA) modulates proliferation and decidualization of endometrial stromal cells.^15^

In addition, Yu et al. showed that overexpression of miR‐375 during decidualization of endometrial stromal cells in vitro significantly reduced the expression of PRL, IGFBP1 and another decidualization marker, namely forkhead box protein O1 (FOXO1).^10^ Furthermore, metastasis‐associated lung adenocarcinoma transcript 1 (MALAT1), a long non‐coding RNA, was found to improve the decidualization of ESCs via binding with miR‐498‐3p, which resulted in the upregulation of histone deacetylase 4 (HDAC4) and maintained cells viability.^16^ However, Liang et al. showed that MALAT‐1 increases during endometriosis and might promote the proliferation and migration of endometriotic stromal cells in a miR‐200c‐dependent manner.^17^ Therefore, the current progress in studies on the interaction between long ncRNAs, small non‐coding RNA and coding RNA (i.e. lncRNA‐miRNA‐mRNA axis) is essential to identify novel targets for combating diseases associated with endometrium.

In this study, for the first time, we have tested the influence of β‐LG on equine EPCs. The main objective of the research was to analyse the potential antioxidant activity of β‐LG and correlate it with the expression of PRL and insulin‐like growth factor–binding protein‐1 (IGFBP1). Moreover, we were interested in whether β‐LG may affect the EPCs' viability. Based on the expression profile of essential biomarkers, we proposed a potential molecular axis of action. Our investigation may contribute to revealing the mechanism in which β‐LG at the molecular level regulates regeneration and functional differentiation of endometrium.

## 2 MATERIALS AND METHODS

### 2.1 Experimental culture conditions

The biological activity of β‐LG was tested using EPCs established at passage 4. Cells were isolated accordingly to the protocol published previously.^8^ The cultures were maintained in a complete growth medium (CGM) composed of medium (DMEM/F12, Sigma‐Aldrich/Merck, Poznan, Poland) supplemented by 15% of foetal bovine serum (Sigma‐Aldrich/Merck, Poznan, Poland) and 1% of antibiotics (Pen Strep, Sigma‐Aldrich/Merck, Poznan, Poland). The cells were cultured in aseptic conditions at 37°C with humidity established at 95% and CO_2_ at 5%. The culture media were changed every 2 days to maintain the proper condition of cells and growth. The EPCs cultures were detached from culture dishes using StableCell™ Trypsin solution sigma (Aldrich/Merck, Poznan, Poland).

For the experiment, cells were plated into 6‐well culture dishes at 100,000 cells/well. The cultures were divided into four groups: (i) control (CTRL)—maintaining the regular condition of growth, cultured in CGM; (ii) treated with β‐LG; (iii) with induced oxidative stress (OS); (iv) with induced oxidative stress and treated with β‐LG. To induce oxidative stress, EPCs cultures were treated with 100 μM of H_2_O_2_ in CGM for 2 h in a 5% CO_2_ incubator at 37°C. Experimental cultures were treated with 5 mg/mL of β‐LG for 48 h under oxidative stress and normal culture conditions. The β‐LG was dissolved in CGM.

### 2.2 Evaluation of β‐LG influence on EPCs cytophysiology—Oxidative status, viability and morphology

The influence of β‐LG on oxidative status and viability was determined using Guava® Muse® Cell Analyser (Sigma‐Aldrich/Merck, Poznan, Poland). After the experiment, the cultures were trypsinized for analysis. The intracellular accumulation of ROS was evaluated using Muse® Oxidative Stress Kit (Muse®: cat.number: MCH100111, Poznań, Poland). In turn, the viability of the cells was analysed using the Muse™ Annexin V & Dead Cell Kit (Merck®; cat. no.: MCH100105, Poznań, Poland). Cells were stained according to protocols provided by the manufacturer (Luminex/Merck, Poznan, Poland) using parameters described in detail previously.^18^ Between the measurements, each sample was vortexed and protected from light.

Morphological and ultrastructural changes in experimental cultures were evaluated using a confocal microscope under magnification 630× (Leica TCS SPE, Leica Microsystems, KAWA.SKA Sp. z o.o., Zalesie Gorne, Poland). The analysis was aimed at determining nuclei and mitochondrial network integrity, as well as cytoskeleton development. The staining procedure was described in detail previously.^8^ Obtained images were analysed with Fiji software (ImageJ 1.52n, Wayne Rasband, National Institute of Health, Bethesda, MD, USA), while the number of mitochondria was assessed using MicroP software (ver. 1.1.11b, Biomedical Image Informatics Lab, Taipei City, Taiwan (R.O.C.) Institute of Biomedical Informatics, National Yang‐Ming Chiao Tung University) powered by MATLAB (version R2010b, The Math Works, Natick, MA, USA).

### 2.3 Evaluation of β‐LG influence on the transcriptome in EPCs—Reverse transcription‐quantitative polymerase chain reaction (RT‐qPCR)

Total RNA was isolated by the phenol‐chloroform method and extracted with Extrazol® (Blirt DNA, Gdansk, Poland). Obtained RNA was purified from genomic DNA using a PrecisionDNAse kit (Primerdesign, Blirt DNA, Gdansk, Poland).

For gene expression analysis, total RNA (500 ng) was transcribed into cDNA using Tetro cDNA Synthesis Kit (Bioline Reagents Limited, London, UK). In turn, for the detection of non‐coding RNA, the cDNA was synthesized using the Mir‐X™ miRNA First‐Strand Synthesis Kit (Takara Clontech Laboratories, Biokom, Poznań, Poland) using 375 ng RNA. The qPCR was performed using SensiFast SYBR & Fluorescein Kit (Bioline Reagents Ltd.) in a total volume of 10 μL, while the concentration of primers in each reaction was 400 nM. The RT‐qPCR was performed accordingly to the instructions of the manufacturers. The purification and reverse transcription were performed in T100 Thermal Cycler (Bio‐Rad, Hercules, CA, USA), while real‐time detection of transcripts accumulation was in CFX Connected Real‐Time PCR Detection System (Bio‐Rad, Hercules, CA, USA). The list of used primers with the sequences is presented in Table S1. The obtained data were normalized using the RQ_MAX_ algorithm described previously.^8^, ^19^

### 2.4 The influence of β‐LG on the expression of proteins associated with decidualization—Western blot

The protocol of protein detection was published previously. Here, cultures were lysed using an ice‐cold RIPA buffer containing 1% protease and phosphatase inhibitor (Sigma‐Aldrich/Merck, Poznan, Polska). As previously, protein concentration in the samples was assessed using the Bicinchoninic Acid Assay Kit (Sigma‐Aldrich/Merck, Poznan, Poland). For SDS–PAGE separation, 14 μg of protein was used. The condition of electrophoresis, transfer and blotting remained unchanged. The details of antibodies used for the reaction are listed in Table S2.

### 2.5 Statistical analysis

The results obtained in the study are presented as the mean with standard deviation (±SD). The values included in the analysis resulted from at least three technical repetitions/measurements. The population data's normality was determined using the Shapiro–Wilk test, while Levene's test assessed the equality of variances. Tests were performed using STATISTICA 10.0 software (StatSoft, Inc., Statistica for Windows, Tulsa, OK, USA). The comparative statistics included a one‐way analysis of variance with Tukey's post hoc test. Calculations were performed using GraphPad Software (Prism 8.20, San Diego, CA, USA). Differences with a probability of *p* < 0.05 were considered significant.

## 3 RESULTS

### 3.1 β‐LG has an antioxidant effect on equine endometrial progenitor cells

**FIGURE 1:**
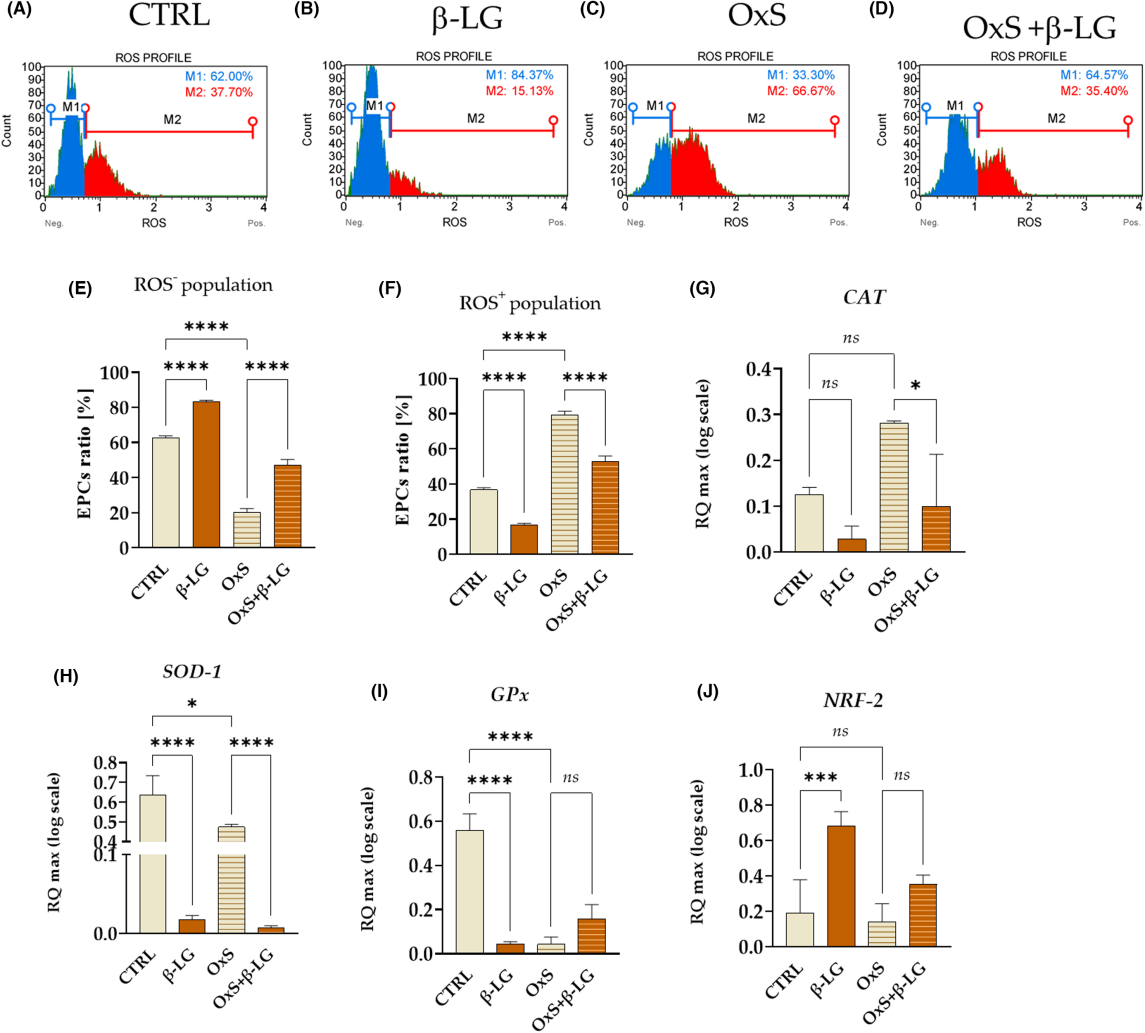
The results of the oxidative status evaluation in endometrial progenitor cells (EPCs) treated with β‐lactoglobulin (β‐LG) under control conditions (CTRL) and oxidative stress (OxS). The distribution of cells was determined based on the intracellular accumulation of reactive oxygen species (ROS) and showed on representative histograms (A–D). The gates on the histogram indicate the distribution of EPCs into ROS‐negative (M1 gate, ROS^−^) and ROS‐positive populations (M2 gate, ROS+). The comparative analysis with statistics was performed on the results obtained from three cytometric measurements (E,F). The study also included the comparison of transcript levels for catalase (CAT; G), superoxide dismutase 1 (SOD ‐1; H) glutathione peroxidase (GPx; I) and nuclear factor erythroid 2‐related factor (NRF‐2; J). Comparative analysis was performed on results obtained from three independent tests. The data are presented as columns with bars mean ± SD. The significant difference was indicated with an asterisk, that is *p‐value < 0.05 and ****p‐value < 0.0001, while *ns* symbol refers to non‐significant differences.

To test whether β‐LG exerts an antioxidant effect on equine EPCs, we first analysed its impact on intracellular ROS accumulation under control and oxidative stress conditions. The β‐LG was tested in vitro at a 5 mg/mL concentration, established previously based on preliminary screening assays. At this concentration, β‐LG significantly affected the cellular metabolism of EPCs (data not shown). The oxidative stress was successfully induced in EPCs by H_2_O_2_ in a concentration of 100 μM (Figure 1C). ROS levels in the H_2_O_2_‐induced group (OxS) increased significantly compared to the control culture (CTRL; Figure 1A,C,E,F). Notably, β‐LG‐treated cells were characterized by a significant reduction in ROS production (Figure 1B,D–F). Consequently, β‐LG showed a protective effect against H_2_O_2_‐induced oxidative stress in EPCs (Figure 1D,E).

Furthermore, antioxidant properties of β‐LG were assessed based on its modulatory effect on mRNA levels measured for genes coding catalase (CAT; Figure 1G), superoxide dismutase 1 (SOD ‐1; Figure 1H), glutathione peroxidase (GPx; Figure 1I) and nuclear factor erythroid 2‐related factor (NRF‐2; Figure 1J). Analysed transcripts were decreased or remained unchanged in EPCs after β‐LG treatment. This expression profile obtained for genes coding essential antioxidant enzymes was noted in standard culture conditions and under oxidative stress (Figure 1G–J).

Overall, the obtained data showed that β‐LG decreases the accumulation of ROS, accompanied by reduced mRNA expression for SOD‐1 both in normal conditions and under oxidative stress. Simultaneously β‐LG modulates transcript levels for CAT, GPx and NRF‐2. The mRNA expression for CAT in EPCs maintained in control conditions was not changed in response to β‐LG stimulation. However, the accumulation of GPx transcripts was lowered simultaneously with increased mRNA levels for NRF‐2. In turn, transcript levels for CAT were significantly increased under oxidative stress after β‐LG treatment, while GPx and NRF‐2 levels remained unchanged.

### 3.2 β‐LG regulates the viability of endometrial progenitor cells

**FIGURE 2:**
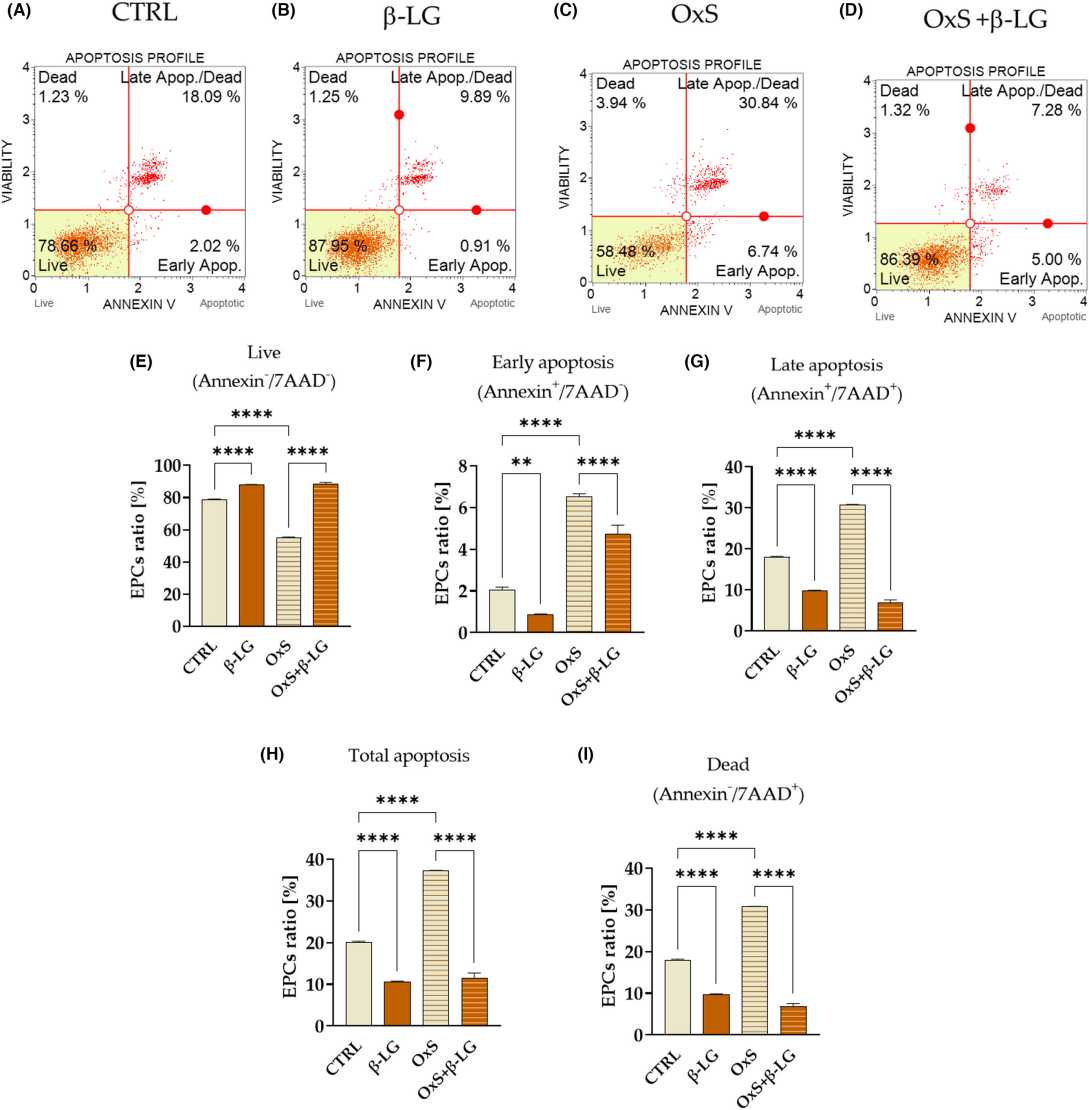
The analysis results of the viability and apoptosis profile modulated by β‐lactoglobulin (β‐LG) in control conditions (CTRL) and under oxidative stress (OxS). Representative dot plots show the distribution of cells based on their cellular health status and staining with Annexin V/7AAD (A–D). The lower left quadrant on schemes shows viable cells(AnnexinV^−^/7‐AAD^−^), while the lower right quadrant represents early apoptotic cells (Annexin V^+^/7‐AAD^−^). The upper right quadrant shows late apoptotic cells (7‐AAD^+^/Annexin V^+^), while the left quadrant shows necrotic cells(7‐AAD^+^/Annexin V^−^). The statistical analysis included the evaluation of live cells (E), apoptotic (F,G,I) and necrotic cells (H). Columns with bars in comparative statistics represent mean ± SD. The significant difference was indicated with an asterisk, that is **p‐value < 0.01 and ****p‐value < 0.0001.

We determined the distribution of EPCs based on Annexin V and 7‐AAD staining to analyse the influence of β‐LG on cellular viability. The analysis of the apoptosis profile (Figure 2) revealed that β‐LG exerts an anti‐apoptotic effect, significantly increasing the viability of cells and simultaneously decreasing the number of apoptotic cells (Figure 2B,D–I). The study showed that β‐LG reduces apoptosis, influencing the elimination of cells with the phenotype of early and late apoptotic/dead cells (Figure 2A–D,F–I). Obtained data showed that β‐LG abolished the negative effect of oxidative stress on the viability and apoptosis profile of cells (Figure 2C,D,E–I).

**FIGURE 3:**
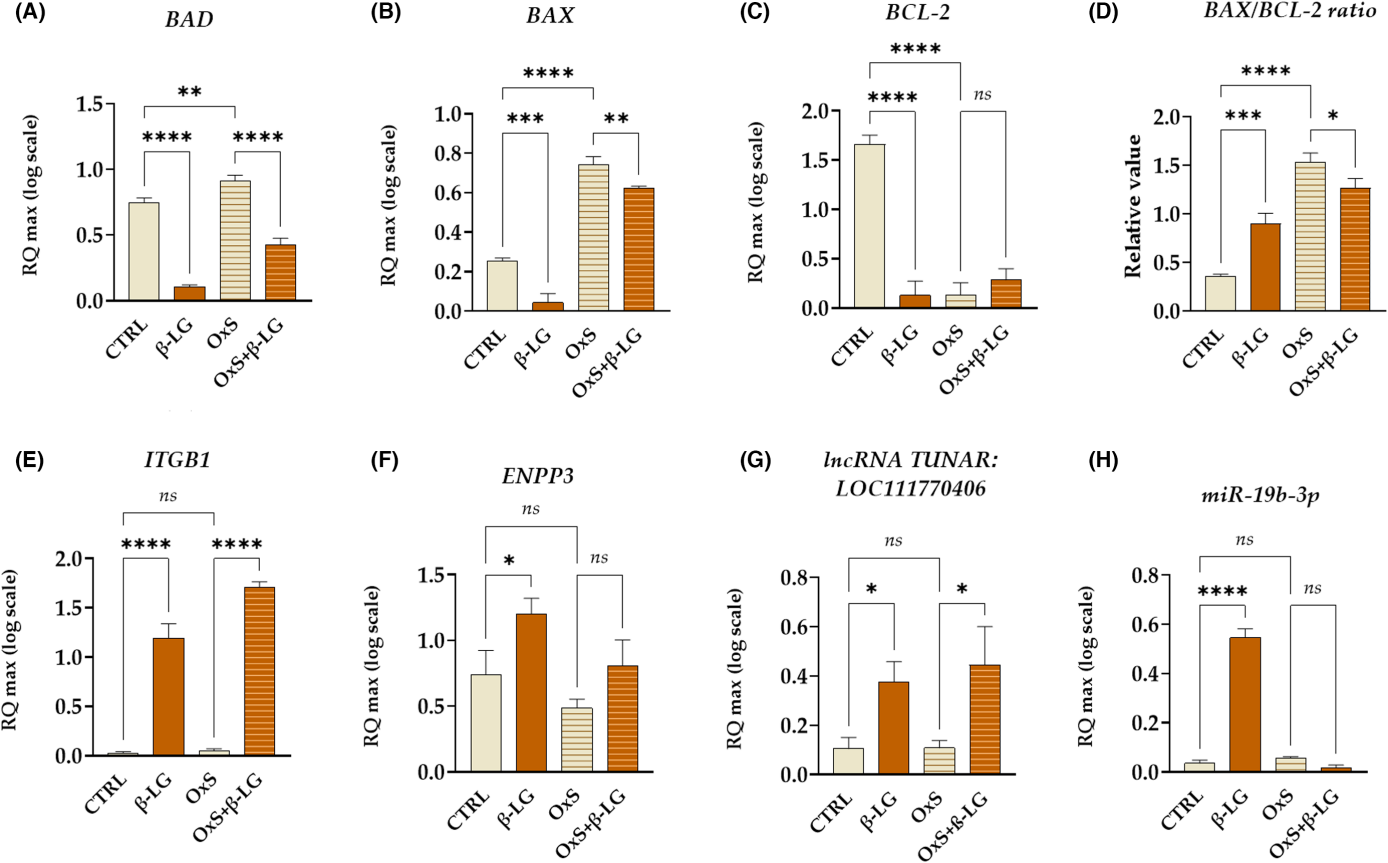
Influence of β‐lactoglobulin (β‐LG) on transcripts associated with cellular viability under control (CTRL) and oxidative stress (OxS) conditions. The analysis included the evaluation of transcripts from the B‐cell lymphoma 2 (BCL‐2) family, including BAD (BCL‐2 associated agonist of cell death, (A) BAX (BCL‐2 associated X‐protein, (B) and BCL‐2. (C) The BAX and BCL‐2 were compared to evaluate BAX/BCL‐2 ratio. (D) The study included measurement of transcripts for ITGB1 (Integrin beta‐1, (E) ENPP3 (ectonucleotide pyrophosphatase‐phosphodiesterase 3, (F) as well as long non‐coding RNA TUNAR (TCL1 upstream neural differentiation‐associated RNA, LOC111770406, (G) and small non‐coding RNA, that is miR‐19b‐3p (H). Columns with bars in comparative statistics represent mean ± SD. The significant difference was indicated with an asterisk, that is *p‐value < 0.05, **p‐value < 0.01, ***p‐value < 0.001 and ****p‐value < 0.0001, while *ns* symbol refers to non‐significant differences.

The levels of pro‐apoptotic regulators, that is BAD (Figure 3A) and BAX (Figure 3B), were also decreased in EPCs treated with β‐LG under standard conditions (CTRL) and H_2_O_2_‐induced (OxS). However, simultaneously the transcript levels of anti‐apoptotic BCL‐2 were significantly reduced in EPCs after β‐LG treatment in standard culture conditions. Moreover, the mRNA levels for BCL‐2 were significantly reduced in cells with induced oxidative stress, and β‐LG treatment did not alter the BCL‐2 expression (Figure 3C). The observed expression profile of BCL‐2 also reflected the BAX/BCL‐2 ratio, indicating an increased amount of pro‐apoptotic transcripts after β‐LG treatment in standard conditions(Figure 3D). Nevertheless, the BAX/BCL‐2 ratio decreased significantly after β‐LG in EPCs with induced oxidative stress (Figure 3D). This result is in line with reduced levels of apoptosis noted after β‐LG treatment (Figure 2A–I). At the same time, we observed an increase in mRNA expression for ITGB1 (Figure 3E) and ENPP3 (Figure 3F) in response to β‐LG. Both molecules are considered essential regulators of endometrial cells' viability and receptivity. Moreover, β‐LG increased transcripts levels for ITGB1 in standard culture conditions and under oxidative stress (Figure 3E). While mRNA levels for ENPP3, regulated by β‐LG, were significantly increased only in standard culture conditions (Figure 3F). Furthermore, the mRNA expression pattern noted for ITGB1 correlated with levels for lncRNA TUNAR (Figure 3G), while ENPP3 transcript levels corresponded with miR‐19a‐3p levels (Figure 3H).

**FIGURE 4:**
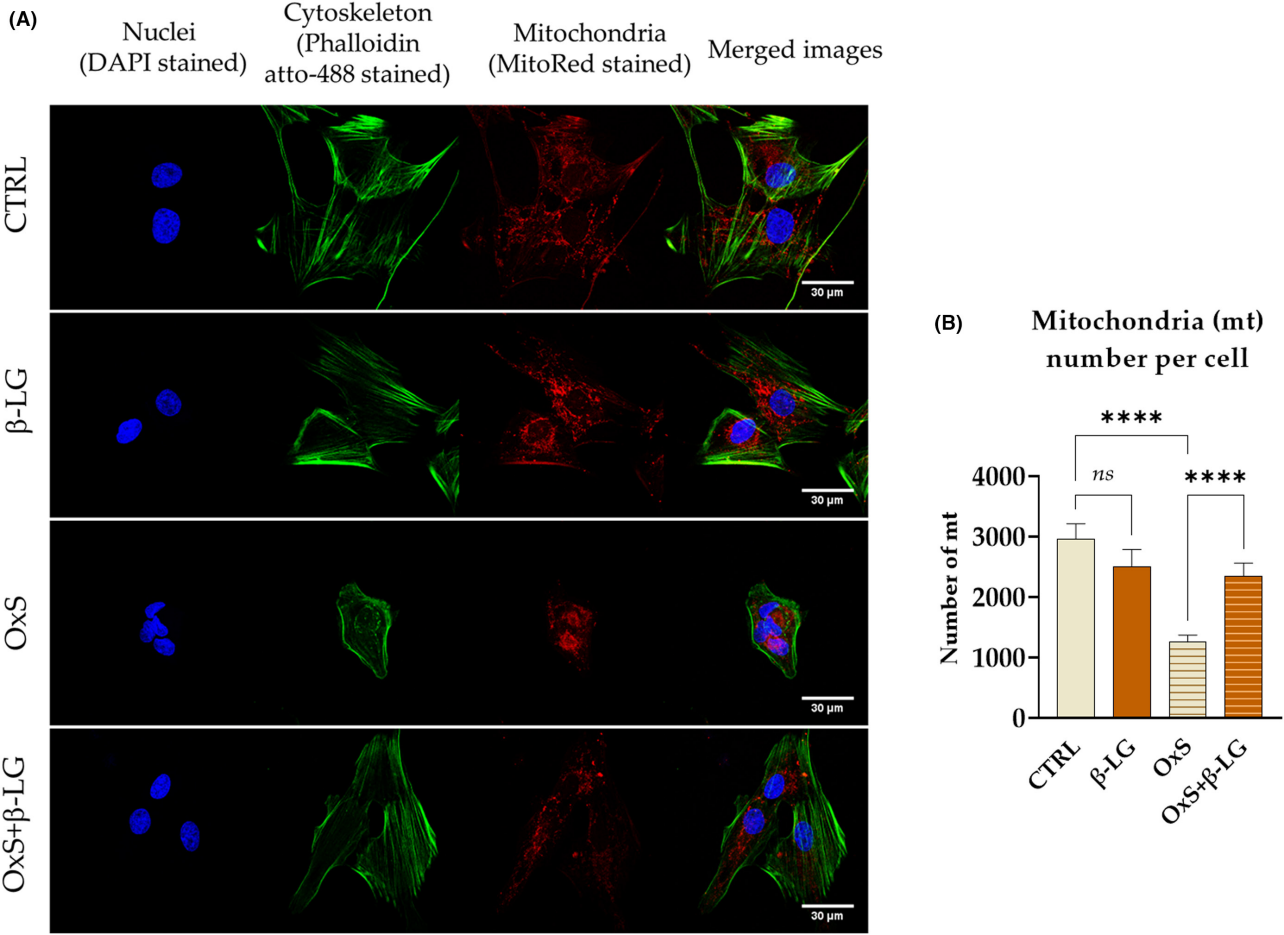
Morphology and ultrastructure of equine endometrial progenitor cells (EPCs) in experimental conditions evaluated under confocal microscopy. The cells were treated with β‐lactoglobulin (β‐LG) under control culture conditions (CTRL) and oxidative stress (OxS). The images were taken under 630× magnification. The scale bar indicated in the figure equals 30 μm. (A) The number of mitochondria per cell was assessed using MicroP software to perform statistical analysis(B) Columns with bars in the graph represent the mean value ± SD. ****p‐value < 0.0001, while *ns* symbol refers to non‐significant differences.

The influence of β‐LG on EPCs viability was also monitored in relation to the architecture of growth and morphology of cells in cultures (Figure 4). We have noted that β‐LG positively affects actin cytoskeleton and mitochondrial network development. β‐LG prevented morphological alterations of EPCs under oxidative stress, such as deterioration of actin cytoskeleton and significant shrinkage of cell bodies (Figure 4A). Moreover, β‐LG counteracted oxidative stress by maintaining appropriate networking of mitochondria and their number within the cell (Figure 4B).

To sum up, the obtained data indicate the positive influence of β‐LG on EPCs' viability. The β‐LG counteracts increased cellular death under oxidative stress, decreasing the overall number of apoptotic cells and reducing levels of pro‐apoptotic transcripts, that is BAD and BAX. At the same time, β‐LG is increasing mRNA expression for ITGB1 and upregulates the expression of lncRNA TUNAR. Moreover, β‐LG allows for maintaining functional growth structure and morphology of EPCs exposed to oxidative stress.

### 3.3 β‐LG modulates the expression of decidualization

**FIGURE 5:**
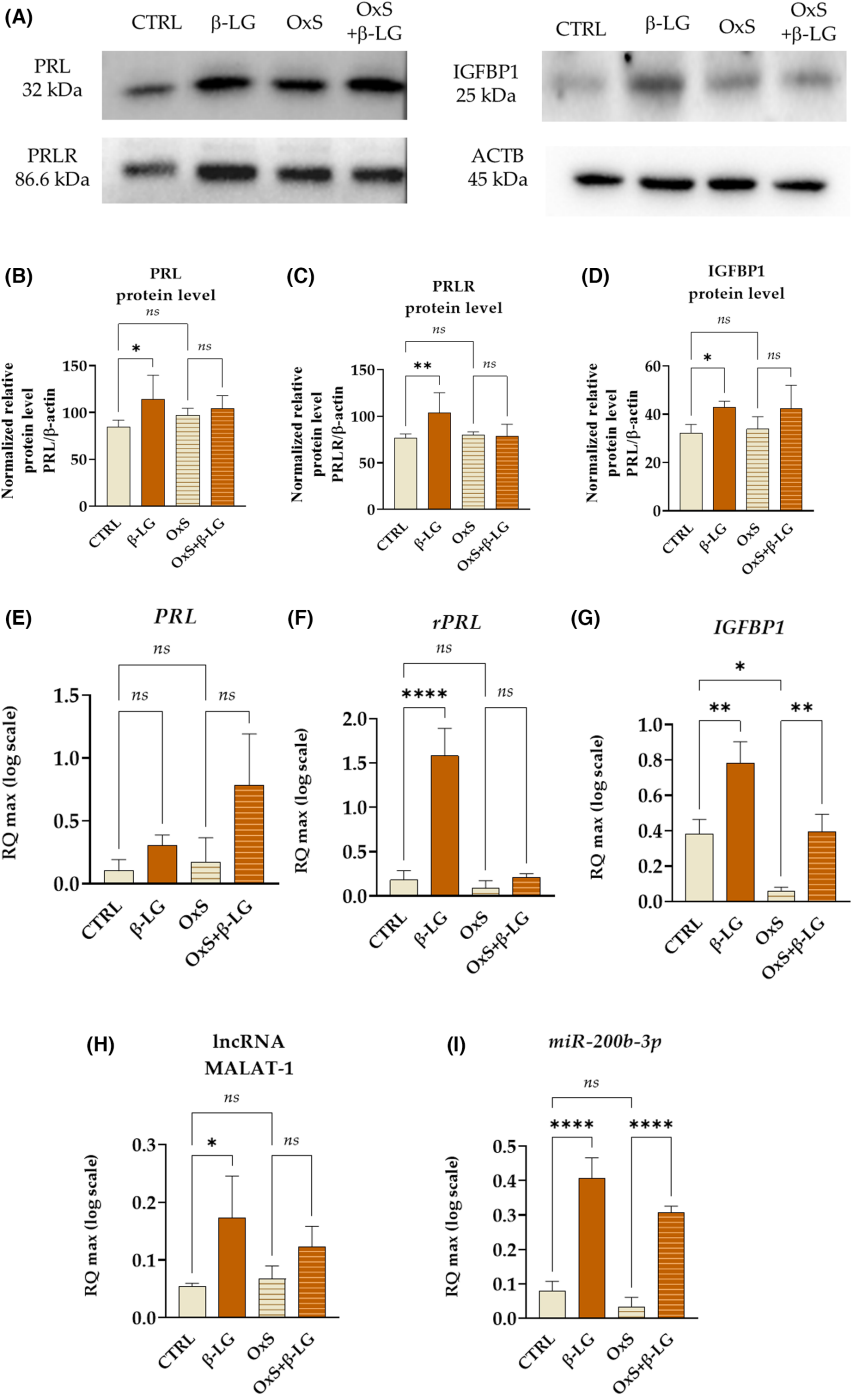
The expression of decidualization markers regulated by β‐lactoglobulin (β‐LG) at protein (a‐d) and transcriptional levels (e‐i). The analysis was performed in control conditions (CTRL) and under oxidative stress (OxS). The prolactin (PRL; A,B,E) and its receptor (rPRL; A,C,F), as well as insulin‐like growth factor‐binding protein 1 (IGFBP1; A,D,G) were tested at protein and mRNA levels. Transcripts for long non‐coding RNA MALAT‐1 (metastasis‐associated lung adenocarcinoma transcript 1; H) and small non‐coding RNA miR‐200b‐3p (I) were also established. Columns with bars in comparative statistics represent mean ± SD. The quantitative statistic was performed from Western blots performed in triplicate. The significant difference was indicated with an asterisk, that is *p‐value < 0.05, **p‐value < 0.01, ***p‐value < 0.001 and ****p‐value < 0.0001, while *ns* symbol refers to non‐significant differences.

Bearing in mind the influence of β‐LG on mRNA levels for ITGB1 and ENPP3, we have decided to test the mRNA levels of decidualization markers, that is PRL and its receptor (rPRL), as well as insulin‐like growth factor‐binding protein 1 (IGFBP1) (Figure 5A–G). Moreover, we have determined the levels of ncRNAs, that is long non‐coding RNA MALAT‐1 and miR‐200b‐3p (Figure 5H,I). Obtained data indicate that β‐LG significantly improves the expression of PRL, rPRL and IGFBP1 at the protein level. However, it does not impact the intracellular accumulation of decidualization proteins under oxidative stress conditions. The oxidative stress did not alter the PRL, rPRL and IGFBP1 protein expression (Figure 5A–D).

The modulatory effect of β‐LG was seen on the transcript level (Figure 5E–I). The mRNA level for rPRL (Figure 5F) and lncRNA MALAT‐1 (Figure 5H) was increased in response to β‐LG only in cells cultured under standard conditions. The β‐LG had no significant impact on the accumulation of PRL transcripts, and only a negligible increase in mRNA expression was observed after β‐LG treatment (Figure 5E). However, the mRNA levels for IGFBP1 were increased in EPCs after β‐LG treatment regardless of oxidative status(Figure 5G), which corresponds with miR‐200b‐3p levels (Figure 5I). Moreover, oxidative stress decreased IGFBP1 mRNA expression (Figure 5G).

In summary, the β‐LG improves the expression of decidualization markers in EPCs at the protein level but does not prevent their decrease under oxidative stress. The significant upregulation of transcripts of both IGFBP1 and miR‐200b‐3p was noted in EPCs after β‐LG treatment, both in normal and oxidative stress conditions, indicating its role in stabilizing the functional phenotype of the cells.

## 4 DISCUSSION

Maintaining endometrium functionality is one of the main challenging aspects of regenerative human and animal medicine.^20^, ^21^ Endometrial progenitors are a unique population of cells that secretory activity and cellular plasticity contribute to endometrial regeneration and repair.^22^, ^23^ Recently, we have characterized equine progenitor cells (Eca EPCs), showing mesenchymal phenotype and multipotent character.^8^ We demonstrated that Eca EPCs disturbed metabolism is associated with intracellular accumulation of ROS. Deteriorated oxidative status of Eca EPCs significantly affects their pro‐regenerative function related to proliferation and multilineage differentiation properties.^8^ Oxidative stress of stromal cells accompanied by tissue inflammation also underlie the pathophysiology of endometriosis.^24^, ^25^ Different agents were applied to target endometrial cell oxidative stress, improve endometrium regeneration and treat endometriosis.^24^, ^26^, ^27^

In the present study, we have tested the potential antioxidant properties of β‐LG using the model of equine EPCs. We have shown that β‐LG mitigates the oxidative stress induced in EPCs by H_2_O_2_, reducing the intracellular accumulation of ROS and simultaneously downregulating transcript levels for CAT, SOD‐1 and GxP.

Previously, β‐LG was recognized as a mild antioxidant with an elusive biological function. β‐LG belongs to lipocalins like Gd, a closely related glycoprotein. While β‐LG is a significant component of ruminant milk, Gd is expressed in the reproductive tissues of primates, including secretory and decidualized endometrium.^28^ Here, we reasoned that β‐LG might regulate the metabolism of EPCs improving their oxidative status. To the best of our knowledge, this concept is original and was not previously studied.

The highest antioxidant activity has β‐LG peptide (58–61), that is LQKW. The antioxidant β‐LG action is correlated with the free sulfhydryl group at Cys‐121.^2^, ^29^ Moreover, Liu et al. showed that Cys‐121, partially buried within the protein core, is oxidized during radical scavenging.^2^ Furthermore, Kim et al. showed that in addition to antioxidant function, β‐LG exerts a senolytic effect on mouse myoblasts triggering its differentiation toward mature cells expressing MYH3 and thus having a potential role in muscle regeneration.^29^

Nevertheless, β‐LG role as a molecule of significant importance in the biology of progenitor cells derived from a unique tissue niche, that is endometrium, has not been studied so far. Here, we have shown that β‐LG reduces the accumulation of ROS in EPCs and may restore disturbed oxidative status. Obtained results are also in line with studies by Pepe et al., who showed that β‐LG ‐derived peptide, that is BRP2 reduced ROS release in intestinal epithelial cells. BRP2 reduced ROS accumulation in cells in a concentration‐dependent manner, exerting a cytoprotective effect against induced oxidative stress.^30^ Pepe et al. also showed that BRP2 upregulates cytoprotective enzymes activating NRF2/ARE pathway.

In our study, we have shown that mRNA expression for NRF2 increases after β‐LG treatment, but significantly only under control conditions. NRF2 is a transcription factor regulating the expression of antioxidant enzyme genes, including CAT, SOD and GPx. It is essential to protect against oxidants, increase cell survival responses and maintain cellular redox homeostasis by regulating ROS production.^31^ Jin et al. previously showed that NRF2 expression in endometrial regenerative cells increases after pretreatment with stromal cell‐derived factor‐1 (SDF‐1), resulting in the enhanced therapeutic potential of the cells.^31^ Furthermore, silencing NRF2 expression in endothelial progenitor cells lowers their survival and resistance to oxidative stress injury.^32^

Here, we also have demonstrated that mRNA levels for CAT, SOD‐1 and GPx decrease in response to β‐LG treatment. Such expression profiles of genes that code primary defence enzymes against oxidative stress may be associated with transcriptome dynamics. Previously, Song et al. showed that administration of lutein in combination with vitamin C improved the antioxidant defence system in 18 eight‐week‐old male Sprague Dawley. However, the administration of antioxidants did not affect mRNA levels of CAT, GPx and glutathione S‐transferase (GST).^33^

In our study, we showed that β‐LG may also influence the viability of EPCs by reducing the number of apoptotic cells. Simultaneously, EPCs after β‐LG treatment had decreased mRNA levels for pro‐apoptotic molecules, that is BAX and BAD and anti‐apoptotic BCL‐2. The BAX/BCL‐2 ratio indicated an accumulation of pro‐apoptotic transcripts in control conditions. At the same time, β‐LG induced anti‐apoptotic signalling under oxidative stress conditions, reversing the impairment of viability induced by H_2_O_2_. The antioxidants may have selective action in terms of the regulation of cell viability. For instance, rutin was shown to act selectively, improving the oxidative status of endometriotic cells, simultaneously inducing their apoptosis by increased accumulation of pro‐apoptotic proteins BAX and caspase 9.^34^

The modulatory activity of β‐LG is used to modify the action of anticancer therapeutics, for example metallodrug oxaliplatin (OXA).^35^ The β‐LG itself can exert an anticancer effect inducing apoptosis of various cancer cells, including lung tumour cells (cell line A549), intestinal epithelial tumour cells (cell line HT29), hepatocellular cells (cell line HepG2) and breast cancer cells (cell line MDA231‐LM2).^36^ However, Ha et al. applying Caco‐2 showed that β‐LG nanoparticles ranging from 98 to 192 nm are uptaken by the cells and localized in the perinuclear area showing no cytotoxic effect.^33^ Collected data confirm the selected bioactivity of β‐LG and its modulatory effect on cell viability.

In our study, we have also shown that β‐LG significantly upregulates the expression of ITGB1—a molecule essential for proper endometrium repair that determines cell–cell and cell‐extracellular matrix interaction. Indeed, β‐LG mitigated unfavourable changes in morphology and growth architecture of EPCs, particularly evident under oxidative stress and concerning disturbance in the actin cytoskeleton, loss of cell volume and shrinkage. The β‐LG‐treated cells maintained intracellular connections and typical growth architecture.

We also noted that EPCs treated with β‐LG show increased mRNA expression for ENPP3. However, a significant difference was noted only in standard conditions, which also correlated with increased levels of miR‐19b‐3p. ENPP3 is a typical ectoenzyme localized to the cell surface and has a crucial role in metabolizing extracellular nucleotides and their derivatives.^37^ However, due to its catalytic activity, ENPP3 also modulates the level of intracellular nucleotide sugars, and most recently, it was recognized as an enzyme modulating endometrial receptivity.^14^, ^37^ The levels of small non‐coding RNA miR‐19b‐3p were not previously established for endometrial progenitor cells. However, it was shown that miR‐19b‐3p promotes proliferation and reduces heterochromatin‐mediated senescence in male germline stem cells.^38^ Moreover, the role of miR‐19b‐3p seems to be essential for the functional differentiation of stem/progenitor cells derived from bone marrow. miR‐19b‐3p may regulate the expression of transcripts with an important role in processes such as cell proliferation, migration and differentiation, including insulin‐like growth factor 1 (IGF‐1) and lncRNA H19.^39^

Furthermore, increased accumulation of ENPP3 transcripts in EPCs after β‐LG treatment correlated with the increased levels of long non‐coding RNA TUNAR (i.e. TCL1 upstream neural differentiation‐associated RNA; here: LOC111770406, an equine homologue). LncRNA TUNAR stimulates the growth but also the proliferation of endometrial stromal cells and thus is a critical regulator of endometrium decidualization. In addition, lncRNA TUNAR was also found to modulate embryo attachment to the endometrial epithelium.^15^

Given the expression profile of the ENPP3‐TUNAR molecular axis characteristic for EPCs treated with β‐LG, we were interested in whether β‐LG modulates the expression of master regulators of endometrial progenitor decidualization, that is PRL, its receptor (PRLR) and insulin‐like growth factor‐binding protein 1 (IGFBP1). We noted that β‐LG impacts and modulates the expression of decidualization‐specific markers in EPCs, improving the intracellular accumulation of PRL and IGFBP1. Obtained results align with the study by Mestre Citrinovitz et al., who showed that resveratrol contributes to endometrial stromal cells' decidualization in vitro, increasing the expression of PRL and IGFBP1.^26^ As a potent antioxidant, resveratrol was also shown to modulate the expression of decidualization markers reversing compromised decidualization elicited by mycotoxin zearalenone (ZEA).^40^

Tightly controlled ROS generation is essential for the differentiation of endometrial stromal cells differentiation. At the same time, PRL signalling is crucial to delay and/or inhibit cellular damage and apoptosis related to oxidative stress in the age‐related degeneration of the retina.^41^, ^42^, ^43^ Moreover, morphological changes accompany decidualization, and besides induction of specific markers, these changes are related to ultrastructural alteration, including actin cytoskeleton organization.^9^, ^44^ However, we did not observe any significant changes caused by β‐LG treatment in the actin cytoskeleton of equine endometrial progenitor cells.

However, the potential positive influence of β‐LG on the functional differentiation of EPCs also confirms the pattern of non‐coding RNA expression, particularly miR‐200b‐3p and lncRNA MALAT‐1 (metastasis‐associated lung adenocarcinoma transcript 1). The levels of miR‐200b‐3p transcripts were upregulated in response to β‐LG treatment, both in cultures with balanced and disturbed oxidative status. This result strongly correlates with mRNA expression for IGFBP1 in EPCs. Obtained results agree with the study by Jimenez et al., who showed that inhibition of miR‐200 expression during decidualization resulted in a decreased expression of IGFBP1 and PRL.^45^ Furthermore, MALAT‐1 was recognized as a molecule facilitating the decidualization of human endometrial stem cells (hESCs) via binding miR‐498‐3p and thus upregulating its target, that is histone deacetylase 4 (HDAC4).^16^ MALAT1 also regulates the viability of hESCs. Its downregulation promoted apoptosis associated with increased BAX/BCL‐2 ratio and suppressed the growth of hESCs.^46^

Nevertheless, MALAT‐1 role in endometrium homeostasis is still elusive, and studies highlighting its role in the development of endometriosis are emerging. For instance, it was shown that MALAT‐1 contributes to hypoxia‐triggered protective autophagy, which is a process that promotes cell survival in endometriosis, reducing apoptosis.^47^, ^48^

To the best of our knowledge, this is the first report showing that β‐LG exerts antioxidant properties and improves the viability of EPCs triggering PRL signalling. The β‐LG also activated posttranscriptional regulation modulating non‐coding transcripts, namely lncRNA MALAT‐1 and TUNAR, as well as miR‐19b‐3p and miR‐200b‐3p.

**FIGURE 6:**
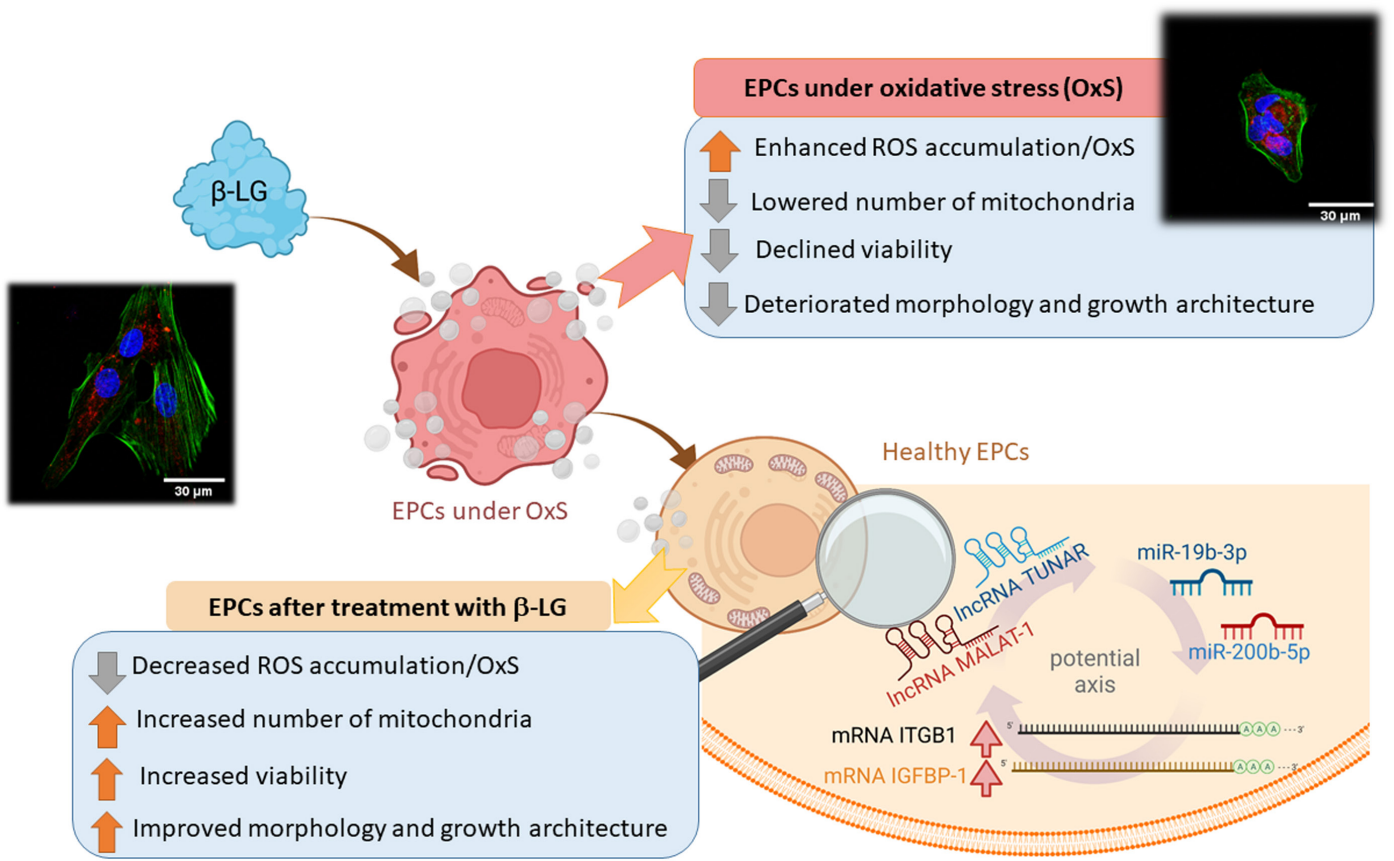
Summary of the most important elements and outcomes of the study.

Our study shed light on the potential application of β‐LG as a new therapeutic option for endometrium regeneration and functional differentiation. The antioxidant activity of β‐LG can benefit the treatment of various fertility‐related endometrial pathologies associated with oxidative stress, including endometriosis (Figure 6).

## AUTHOR CONTRIBUTIONS

**Krzysztof Data:** Conceptualization (equal); data curation (equal); formal analysis (equal); funding acquisition (equal); investigation (equal); methodology (equal); writing – original draft (equal); writing – review and editing (supporting). **Klaudia Marcinkowska:** Investigation (equal); methodology (equal); writing – original draft (equal). **Klaudia Buś:** Visualization (supporting); writing – original draft (supporting). **Lukas Valihrach:** Validation (equal); writing – original draft (supporting); writing – review and editing (supporting). **Edyta Pawlak:** Validation (equal); writing – original draft (supporting); writing – review and editing (equal). **Agnieszka Śmieszek:** Conceptualization (lead); data curation (equal); formal analysis (equal); funding acquisition (equal); investigation (equal); methodology (lead); project administration (equal); resources (lead); supervision (lead); validation (equal); visualization (equal); writing – original draft (lead); writing – review and editing (lead).

## CONFLICT OF INTEREST STATEMENT

The authors declare no conflict of interest.

## Supporting information


**Table S1.** Sequences and characteristics of primers used for RT‐qPCR.

**Table S2.** List of antibodies used for the Western blot.

## Data Availability

The data that support the findings of this study are available from the corresponding author upon reasonable request.
